# How to jointly control a ball trajectory on a moving board: Methodological insights into Motor Learning and Rehabilitation

**DOI:** 10.1371/journal.pone.0334588

**Published:** 2025-10-15

**Authors:** Anaëlle Cheillan, David M. Jacobs, Pedro Passos

**Affiliations:** 1 Faculdade de Motricidade Humana, Universidade de Lisboa, Lisbon, Portugal; 2 Facultad de Psicología, Universidad Autónoma de Madrid, Madrid, Spain; 3 CIPER, Faculdade de Motricidade Humana, Universidade de Lisboa, Lisbon, Portugal; University of Shanghai for Science and Technology, CHINA

## Abstract

Motor (re-)learning can be assessed using various conceptual and methodological frameworks, each of which portrays the learner’s abilities in different ways. The present paper investigates how the direct learning theory can be applied to the assessment of perception-action couplings required for a highly-dimensional joint-action task. Eleven novice dyads were instructed to stand on an unstable surface (BOSU), while jointly manipulating a board with the aim to make a ball roll along a target. Ball and board’s three-dimensional movements were recorded with an 8-camera motion capture system. Linear regression analyses were conducted to examine how task performance – using ball kinematics – and dyadic behaviour – using board kinematics – evolved with practice. Correlation analyses between movement and informational variables were used as a first step to build information spaces. Information spaces are a direct learning tool developed to investigate how the available information is exploited when practicing a new motor task. With practice, dyads reduced the variability in task-irrelevant degrees of freedom, while increasingly coupling the task-relevant degrees of freedom to more optimal informational variables. Finally, information spaces were discussed as a valuable tool for assessing and guiding the re-learning of perception-action in rehabilitation.

## 1. Introduction

Teaching a new (or previously lost) motor skill and assessing the outcomes of (re-)learning processes are important tasks for coaches and therapists in both sports and clinical settings. While these professionals all share the common goal of improving an athlete’s performance or a patient’s activities in daily life, their approaches towards this goal can be based on different, conflicting, theories of motor learning. Conceptual divergences in motor learning should not, however, be overlooked, as they can lead to fundamentally different interpretations of the movement outcomes or disorders observed in athletes or patients. These differences extend to the application of diverse assessment and training methodologies, each of which uniquely influences the learner’s practical abilities [1–3]. Furthermore, unawareness of this plurality of perspectives on motor learning can hinder the development of both original knowledge and innovative practices [2,4].

Traditionally, the concept of learning a new motor skill has been associated with the acquisition of a new motor program internally stored in the brain and reinforced through repetition to develop automaticity in skilled performance [5]. Within this framework, novice performers can repeatedly mimic the one “correct” technique demonstrated by an expert to acquire and consolidate a new motor skill. However, the correct, gold-standard technique achieved by reinforcing cognitive programs through repetition is not the only existing approach to understand expertise.

The ecological dynamical approach of motor control, largely inspired by the pioneers J.J. Gibson [6] and N. Bernstein [7], offers an alternative view of learning and expertise. Rather than reflecting a sophistication of acquired cognitive processes, expert performance results from exploring the relation between information and movement, leading to an improved fit between the actor and environment – or, in Gibson’s terms, an expanded field of *affordances* (i.e., opportunities for action that an actor can detect and use, based on the information available in the environment and their own abilities). Also, instead of depicting the expert as an advanced human-machine capable of executing and replicating an error-free technique, Bernstein introduced the concept of *“repetition without repetition”* to emphasize that movement variability should not be considered as noise to be minimized, but rather as an essential feature of biological organisms, inherently and inevitably involved in the learning process. In this view, experts are self-organized problem solvers who master the many degrees of freedom across their body, task and environment by exploring information-movement couplings and developing coordinative structures [8], which enable them to adapt to constraints and effectively exploit variability in the environment-actor system [9,10]. To sum up, Gibson’s and Bernstein’s work gave rise to a systems perspective of motor learning, where the learner’s coordination dynamics emerge from perception-action couplings (i.e., couplings between the detected information and the produced behaviour) [11] through task-specific interactions between the actor and environment.

From a systems perspective, coaches or therapists could reformulate their ultimate goal as guiding their athletes or patients through a (re-)learning perception-action process. To better understand how new movement solutions emerge from this process, movement can be analysed in relation with the information learners detect and use. Jacobs and Michaels’ direct learning approach [12] offers a theoretical and methodological framework to support this analysis. This approach starts from the assumption that there exist higher-order properties of the environment-actor system and that such properties are specified by detectable information in ambient energy arrays. For instance, the time-to-contact property of an approaching object can be directly perceived using the variable *tau* (i.e., ratio of optical size to the rate of optical expansion) [13], which thus is an information available in the ambient optical array that is detectable by our perception-action system. In this context, learning can be defined as the change in set-up of perception-action systems, where non-optimalities can be reduced in three manners: (1) an improvement in choosing which of the possible perceptions and actions are intended to be actualized (*education of intention*), (2) an improvement in detecting a more useful informational variable, which is to say, a variable that specifies the property intended to be perceived (*education of attention*), and (3) an adjustment in the parameters of the information-movement control law (*calibration*). To investigate how the usage of information in ambient energy arrays changes with practice, methodological tools such as information spaces have been developed by the authors of direct learning theory.

An information space is defined as a space in which each point represents a higher-order property of ambient energy arrays. While there is no single method for constructing an information space, its primary purpose is to map how learners gradually refine their use of available information. As learners practice, their navigation in the information space can be represented as trajectories – or paths – that connect the datapoints. These paths through the space portray change due to learning, defined as the process by which dyads evolve from less useful to more useful (in the sense of task-relevant) informational variables available in the ambient energy arrays. In the direct learning theory, this process of detecting more useful variables with practice to reach better performance is also referred to as *“education of attention”*, and learning is said to be *direct* because the routes navigated through information spaces are guided by information.

The present paper aims to investigate how the direct learning framework can have practical implications in the assessment of performers’ behaviour as they learn a new joint-action task. The joint-action task developed for this study involves a ball-and-board game in which participants are asked to jointly manipulate a board to guide a ball along a circular target path, all while standing on an unstable surface. The protocol choice of using a joint-action task that is challenging for the control of balance arose from the idea of testing a novel approach for the rehabilitation of postural deficits, one that would constrain two actors to behave as a single unit. Also, the task was designed in such a way that it would be comparable to a functional, daily life activity (e.g., jointly carrying and tilting a sofa, loading a truck together, moving a dinner table with guests while avoiding spills from glasses or dishes). Finally, our ball-and-board task seems to be well-suited to an information space analysis, because it was performed by novice participants who may need to refine their perception-action couplings – that is, to navigate within an information space – in order to improve their performance. A notable contribution of this work lies in the application of information-space analysis to a task of considerable complexity and high dimensionality. Indeed, our analyses are expected to extend the information-space methodology, which has previously been applied to lower-dimensional tasks that are performed by individuals [12,14], to a challenging, high-dimensional task performed by dyads. Given our future intention to test our joint-action task as a rehabilitation device, the applicability of the direct learning methodology employed here to study learning behaviour during joint action will also be discussed from a clinical standpoint.

More specifically, the present paper addresses the following research questions:

- *How do dyadic performance and motor behaviour evolve when learning how to jointly move the board to control the ball trajectory?*
- *What information is used when learning how to jointly move the board to control the ball trajectory?*

We first hypothesized that task performance would improve with practice. At the behavioural level, we expected that dyads would increasingly produce board movements that are useful to control the ball trajectory along the target, while decreasingly inducing board movements that are irrelevant to task performance. More precisely, we hypothesized that the board’s roll and pitch rotations – which, if used properly, may induce a circular trajectory of the ball on the board – would be more pronounced at the end of practice, while board movements that are less directly related to the ball trajectories, such as translations, would tend to be eliminated.

To control these board movements, we also hypothesized that dyads would increasingly seek out more useful information as practice progressed. In the language of the direct learning framework, this change in information-movement couplings should manifest itself as navigation within information spaces that is related to task-relevant movements. To summarize our hypotheses, then, we expected that task-relevant degrees of freedom would be increasingly used and that their control would be optimized by coupling them to more useful informational variables, whereas task-irrelevant degrees of freedom would gradually be frozen out.

## 2. Materials and methods

### 2.1. Participants

Twenty-two university level students (eight men and fourteen women; mean age: 21.83 + /- 2.12) participated in this study. Participants were randomly assigned into eleven dyads. Sample size was determined using F-tests (ANOVA, Repeated Measures, Within Factors) for a group and 2 measurements, using parameters such as an effect size of 0.40, an alpha error probability of 0.05, a beta error probability of 0.20 (i.e., a power of 0.80), and a correlation among measures of 0.50. The G*Power software (Universität Düsseldorf, Germany) facilitated this calculation. All participants declared having no history of lower limb injuries, condition affecting postural stability, neurological disorder or visual deficiency. Prior to participation, all participants provided free and informed consent. The study protocol was approved by the Ethics Committee CEIFMH (Conselho de Ética para a Investigação da Faculdade de Motricidade Humana; N° 28/2022).

### 2.2. Ball-and-board device

The ball-and-board device (mass: 2.5 kg; dimensions: 114 x 114 x 8 cm) featured a hexagonal board made of polyurethane (long diagonal: 96 cm). A ring-shaped handle, constructed from a 25-mm polyethylene pipe, was attached to the board so participants could hold it comfortably. Six rigid PVC pipes connected the board to the handle, while six polystyrene rods were secured along the board edge to serve as a ball-stop mechanism. The materials were chosen to ensure the device remained lightweight yet structurally rigid, considering its large size. A 10-cm-wide target path, bordered by two circles (inner radius: 20 cm; outer radius: 30 cm), was drawn on the board, with a target midline similar to road markings serving as a reference. Four equidistant doors marked with paper pins were added along the target path, at the nearest and furthest target points from both participants as well as at the points further to the left and right. The ball used in the task was a reflective marker (diameter: 12.7 mm), while another reflective marker was positioned at the board centre. Additionally, four reflective markers were placed on the edge of the board at the locations where participants held it with their left and right hands. Each participant stood on an unstable BOSU surface, consisting of an inflated rubber hemisphere attached to a rigid platform. For additional postural challenge, the BOSU was placed rounded-side down, with participants balancing on the flat, rigid surface.

### 2.3. Ball-and-board game

**Fig 1:**
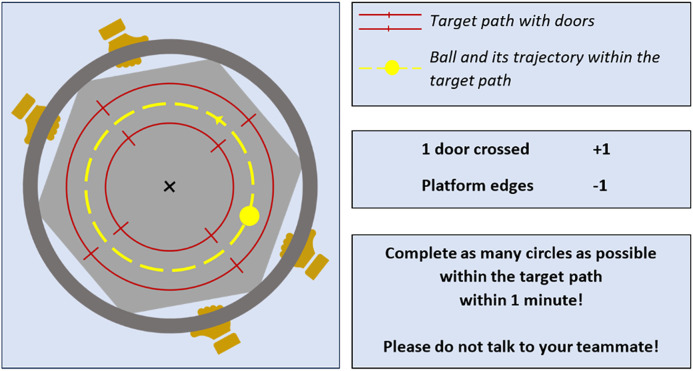
Used apparatus and instructions provided to participants before starting the game. In each dyad, one participant counted the number of crossings, while the other counted the number of ball touches with the board edge.

Participants attended an experimental session consisting of 30 trials. During the experimental session, each dyad – two participants standing barefoot on unstable surfaces (BOSU) – faced each other while jointly manipulating a board over which a ball rolled. For each trial, the participants used a supination grip to hold and move the board, in such a way that the ball completed as many circles as possible in a counterclockwise direction within the target path over a 60-second period (Fig 1). During the trial, one participant tracked the number of doors the ball crossed (i.e., earning 1 point per crossing), while the other counted penalties (i.e., deducting 1 point each time the ball touched the edge of the board). Throughout the session, dyads were updated with their highest score (total crossings minus penalties) for motivational purposes. Instructing dyads to count their scores themselves was only intended to keep them engaged in the game, as performance was later assessed with objectives measures by the experimenter. Any trial in which the ball fell or a participant lost balance and stepped off the BOSU was repeated. Dyads were not allowed to discuss strategies during the experimental session.

Following the recommendations given by participants recruited during pilot tests, the session was divided into two 15-trial sets (hereafter referred to as Set 1 and Set 2), separated by a 5-minute break to minimize fatigue effects. To confirm participants understood the game instructions, they engaged in a brief, unrecorded free-play period (i.e., less than 3 minutes) before the session began.

### 2.4. Data collection and processing

Data collection was conducted using the OptiTrack 8-camera motion capture system (NaturalPoint, Corvallis, Oregon) in conjunction with Motive: Body software (version 2.1.1). Following data collection and labelling, the data were exported at 28 Hz to Excel and subsequently processed in MATLAB (version R2023b, MathWorks Inc., USA). This sample frequency was chosen based on pilot tests and recommendations from previous studies involving a supra-postural task or a joint-action task performed on an unstable surface [15,16]. Using ball and board markers, ball motion relative to the target path and doors was obtained within the board coordinate system. Markers placed on the board’s centre and edges also enabled the recording of translational and rotational movements of the board in the three-dimensional space. The first 3.5 seconds of each trial were discarded to offset inconsistencies in the onset of the ball movement, resulting in a continuous 60-second trajectory (i.e., 1,680 data points). Prior to analysis, occasional missing data were handled using the MATLAB fillmissing interpolation function. As derivatives of variables of interest were considered, a low-pass 6-order Butterworth filter with a cut-off frequency of 1 Hz was applied to all time series.

### 2.5. Data analysis

In the following analyses, the statistical *p*-value threshold was conventionally set at **p* *< 0.05.

#### 2.5.1. Ball kinematic analyses.

Ball kinematics were analysed to investigate task performance. Both speed and accuracy abilities were required in our task where participants were instructed to guide the ball towards as many doors as possible within the target path over a 60-second period. To fully capture task performance, we considered three complementary metrics. First, the number of laps (i.e., how many full circles the ball completed) reflected speed. Second, the within-trial average distance between the ball and the target midline (i.e., the average deviation from the dashed line) was related to accuracy. Third, the number of crossings (i.e., how many doors the ball passed through) served as a global performance score encompassing both speed and accuracy aspects. Relationships between performance variables and practice (i.e., number of trials) were analysed with linear regressions.

#### 2.5.2. Board kinematic analyses.

**Fig 2:**
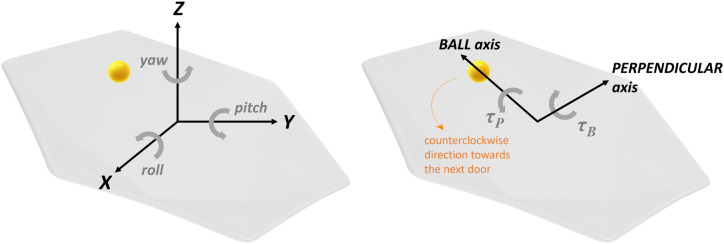
Coordinate systems used for board kinematic analyses *(Left)* and information-movement analyses *(Right).* *Left* panel: Translational and rotational board movements were analysed along and around the *X-*, *Y-* and *Z-*axis in the three-dimensional space originating at the board centre. *Right* panel: The centring torque (**τ**_**B**_) and the forward torque (**τ**_**P**_), which participants controlled to achieve the task, were analysed with a two-dimensional coordinate system originating at the board centre. This coordinate system is defined by the ball axis and its perpendicular axis, and it varies dynamically with the ball position.

Board kinematics were analysed to investigate dyadic behaviour, because the board jointly held by the participants can be viewed as the effector space. Translational and rotational movements were inspected in all three dimensions. For this purpose, the *X*-axis was defined as the left-right axis as seen from the perspective of the participants, the *Y*-axis as the forward-backward axis, and the *Z*-axis as the up-down axis (Fig 2, *Left* panel). Rotations around these respective axes were referred to as roll, pitch and yaw. For each dyad and trial, the standard deviations of both translation along and rotation around each axis were computed, based on the assumption that variability in the board movement reflects the extent to which each movement type is used. Averaging these outcomes across dyads and trials provided insight into how board motion evolved with practice. Finally, linear regression analyses were performed to investigate the relationship between board motion and amount of practice.

#### 2.5.3. Correlation analyses between movement and informational variables.

This analysis section consists of identifying informational variables on which participants rely to produce the movements required to successfully perform the ball-and-board task. One first step to carry out these analyses is to select the movement variables that participants control. Arguably, the most relevant movements for the task are the board’s rotations around the axes that are parallel to the main surface of the board and pass through its centre, because these rotations change the slope of the board and hence the trajectory of the ball. For this part of the analysis, we made the simplifying assumption that this rotation was the only relevant movement of the board. In addition, we used a coordinate system that depends on the ball position and that hence changes with the ball position (Fig 2, *Right* panel). One axis of rotation, referred to as ball axis, was the axis through the midpoint of the board and through the ball. The second axis, referred to as perpendicular axis, was the one through the midpoint of the board and parallel to its main surface, but perpendicular to the first axis. From the registered kinematics, we computed the torques that the dyads applied around these two axes of rotation. The torque applied around the perpendicular axis that lifts the board along the ball axis is referred to as **τ**_**B**_. The other torque, which is applied around the ball axis and therefore lifts the board along the perpendicular axis, is hereafter referred to as **τ**_**P**_. The use of **τ**_**B**_ and **τ**_**P**_ allows for a continuous analysis of the forces that control the board’s roll and pitch rotations relative to the ball position, thereby grounding the analysis in the context of the task – controlling the ball trajectory on the board. For clarity, the right panel of Fig 2 shows both torques, **τ**_**B**_ and **τ**_**P**_, within the coordinate system depending on the ball position (i.e., the coordinate system defined by the ball and perpendicular axes).

Once **τ**_**B**_ and **τ**_**P**_ are claimed to be the relevant action parameters of the task, the next step of the analysis is to determine which information is used for the control of those variables. Intuitively, we set up a list of 16 candidate informational variables that would best predict the participants’ action parameters, namely, the zero-, first- and second-derivatives of i) the distance between the ball and its closest point on the target midline, ii) the distance between the ball and the board centre, iii) the angular ball position around the centre, and iv) the angle between the ball’s direction of movement and the tangent to the target midline, as well as the zero- and first-derivatives of v) the board angle lifting the ball axis and vi) the board angle lifting the perpendicular axis. Please note here that the second-derivatives of those two last variables were not considered, because the angular board acceleration and the applied torque in the same direction are likely to be quite similar, given the relatively lightweight nature of the board.

The final step of this section is to conduct correlation analyses between each candidate informational variable and both action parameters. A visuomotor delay of 0.1 second was taken into account in the correlations [14,17]. For each torque, the three informational variables with greatest absolute correlation values were retained as best-predicting variables for building information spaces. The normalized versions of these variables were called **V1**, **V2** and **V3** in the information space related to torque **τ**_**B**_, and **W1**, **W2** and **W3** in the information space related to torque **τ**_**P**_.

#### 2.5.4. Information space analyses.

Because the key aspects of the motor skills in our task could be described by two action parameters (i.e., torque **τ**_**B**_ and torque **τ**_**P**_), two information spaces were built in the present paper. In both information spaces, each point represents a higher-order property of ambient energy arrays. This higher-order property is a compound informational variable defined by a linear combination of the three best-predicting component variables that were selected at the previous stage of analysis. This compound variable was called ***V*** for the information space related to torque **τ**_**B**_ and ***W*** for the information space related to torque **τ**_**P**_, so that:


V = i(V1) + j(V2) + (1 – i – j)(V3) (1)

W = i(W1) + j(W2) + (1 – i – j)(W3) (2)

In Equations 1 and 2, *i* and *j* are weight coefficients that indicate the contribution of each component to the compound variable. Graphically, these equations restrict the 3-dimensional information space defined by the axes **V1**, **V2**, **V3**, or **W1**, **W2**, **W3**, to a 2-dimensional information space defined by the axes *i* and *j*. In this 2-dimensional information space, the evolution of the used compound variables was illustrated for each dyad with two datapoints of coordinates (*i*, *j*), obtained for Set 1 and Set 2. In each set and for each dyad, the coordinates that were used were the ones that best predicted the respective torques. In both information spaces, learning paths were traced by connecting the datapoints from Set 1 and Set 2 for all 11 dyads. By depicting how the use of informational variables – themselves correlated with torques that induce task-relevant board movements – evolves with practice, information spaces can shed light on the learning process of perception-action couplings during motor skill practice.

## 3. Results

The present section addresses: 1) the evolution of task performance using ball kinematic analyses, 2) the evolution of dyadic behaviour using board kinematic analyses, 3) the usage of informational variables analysed with the correlation between those variables and the action parameters (i.e., the torques that induce task-relevant board movements), and 4) the portrayal of learning as trajectories in information spaces.

### 3.1. Ball motion (task performance level)

**Fig 3:**
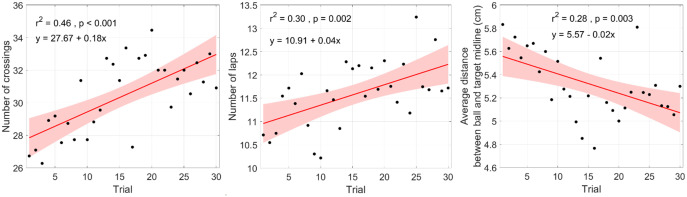
Evolution of task performance over trials. Performance was measured with the number of crossings (*Left*), the number of laps (*Middle*) and the average ball-target distance (*Right*). The presented values are averages across dyads. Also shown are regression equations and associated correlations values and significance levels.

The evolution of task performance over practice was assessed with the number of crossings, the number of laps and the average distance between the ball and the target midline (Fig 3). As also indicated in the figure, linear regressions revealed that the amount of practice (i.e., trial number) was a significant predictor for each of these variables (*F* = 23.68, **F* *= 12.27, and **F* *= 10.70, respectively; all *p*s < .01). Taken together, the increased number of crossings, the increased number of laps and the decreased ball-target distance indicate that performance improved, both in terms of speed and in terms of accuracy.

### 3.2. Board motion (behavioural level)

**Fig 4:**
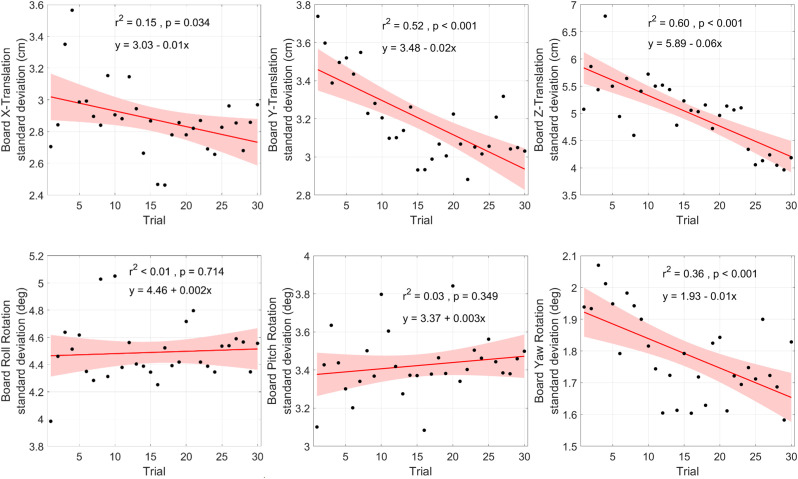
Evolution of dyadic behaviour over trials. The behavior of dyads was measured by the variability of board translations (*Top*) and rotations (*Bottom*) along and around the *X-*axis (*Left*), *Y-*axis (*Middle*) and *Z*-axis (*Right*). The presented values are averages across dyads. Also shown are regression equations and associated correlations values and significance levels.

The board motion analysis allowed us to track the evolution of dyadic behaviour. Fig 4 represents the variability of the board translation and rotation in all three dimensions as a function of trial number. As shown in the top panels of the figure, the association between trial number and variability in board translation was significant for all three axes (**F* *= 4.95, **F* *= 29.73, and **F* *= 41.56, respectively; all *p*s* *< .05). The lower right panel shows that the association was also significant for rotation around the *Z*-axis (i.e., yaw rotation; *F* = 15.74, *p* < .001). The negative slopes in the regression equations indicate that the variability in these four parameters decreased with practice. Although the slopes were positive for the rotations around the *X*- and *Y*-axis (roll and pitch rotation), the regression models were not significant for these variability measures (*p*s > .34). To summarize, we observed a stabilization of the board with practice for the translations along all axes as well as for the yaw rotation. In contrast, the variability in the roll and pitch rotations could not be predicted by the amount of practice.

### 3.3. Informational basis of behaviour

This subsection inspects which informational variables best predict the task-relevant action parameters – namely, the torques inducing a lift of the ball axis (**τ**_**B**_) and the perpendicular axis (**τ**_**P**_), as hypothesized and largely confirmed in the previous subsection. Table 1 presents the average correlations across dyads between 16 candidate informational variables and each torque.

**Table 1:** Pearson’s *r* correlations between 16 candidate informational variables and the torques lifting the ball and perpendicular axes.

Candidate Informational Variables	τ_B_	τ_P_
Distance Ball – Target midline	– 0.15	– 0.14
* First derivative*	– 0.13	0.10
* Second derivative*	0.16	0.06
Distance Ball – Board centre	– 0.24	– 0.09
* First derivative*	– 0.19	**0.36**
* Second derivative*	**0.58**	0.20
Angular ball position (around the board centre)	0.01	– 0.03
* First derivative*	– 0.16	– 0.24
* Second derivative*	– 0.13	0.11
Angle Ball’s direction – Tangent to target midline	0.15	– 0.29
* First derivative*	**– 0.48**	– 0.13
* Second derivative*	– 0.10	0.11
Board angle lifting the ball axis	**– 0.40**	– 0.08
* First derivative*	– 0.35	**0.39**
Board angle lifting the perpendicular axis	– 0.11	**– 0.31**
* First derivative*	0.13	0.05

*Note*. For both torques, the three best-predicting informational variables are shaded in light, medium and dark grey. In terms of Equations 1 and 2, the variables shaded in light grey correspond to **V1** and **W1**, those shaded in medium grey to **V2** and **W2**, and those shaded in dark grey to **V3** and **W3**.

The three best predictor variables for **τ**_**B**_ were: the second derivative of the distance of the ball to the board centre (or outward acceleration; **r* *= 0.58), the first derivative of the angle between the direction of the ball and the tangent of the target midline (**r* *= – 0.48) and the board angle lifting the ball axis (**r* *= – 0.40). To further explain the meaning of the first correlation, the more the outward acceleration increases, the more the centring torque (**τ**_**B**_) increases. This outward acceleration of the ball should not be interpreted as a physical consequence of the torque. If it were, applying a centring torque would instead increase the inward acceleration of the ball. Therefore, the outward acceleration should be understood as an informational variable used to regulate the torque (**τ**_**B**_): if the ball moves outwards, the torque is applied to steer it back to the board centre. The second correlation implies that a greater rate of change of the ball-tangent angle (where higher angle values indicate a further outward direction of the ball) would lead to a decreased torque (**τ**_**B**_). Finally, an increased board lift in the direction of the ball axis (i.e., around the perpendicular axis) goes together with a lower centring force acting on the board, which in turn allows the ball to drift away from the board centre. In other words, the more the board angle increases to guide the ball towards the centre, the less centring torque (**τ**_**B**_) is applied. Notably, this correlation could not plausibly be negative if the board lift were a physical consequence of the applied torque. Similarly, a negative correlation was observed between the board lift in the direction of the perpendicular axis and torque **τ**_**P**_ (**r* *= – 0.31): the greater the board angle that drives the ball forwards, towards the next target door, the less forward torque (**τ**_**P**_) – which also steers the ball in that direction – is applied. Because both correlations were negative, the board angles in the direction of the ball and perpendicular axes were not consequences of the torques that were responsible for lifting the board in the direction of these respective axes, but served as informational variables used to regulate these torques.

Besides the board angle lifting the perpendicular axis, torque **τ**_**P**_ was best predicted by the two following informational variables: the first derivative of the distance of the ball to the board centre (or outward velocity; **r* *= 0.36) and the first derivative of the board angle in the direction of the ball axis (**r* *= 0.39). The positive correlation between **τ**_**P**_ and the ball’s outward velocity indicates that an increased forward torque (**τ**_**P**_) that guides the ball in the direction of the next door would be applied when the ball accelerates outwards. Because torque (**τ**_**P**_) only affects the ball kinematics in the backward/forward direction, the ball’s outward velocity served as information for torque control. As for the last-mentioned correlation, it reveals that forward torque (**τ**_**P**_) is more prone to increase when the angular velocity of the board responsible for centring the ball also increases. Again, this board angular velocity that centres the ball is independent of the torque that drives the ball forwards and should therefore be understood as an informational variable rather than a physical consequence of the applied torque.

### 3.4. Learning behaviour as paths in information spaces

**Fig 5:**
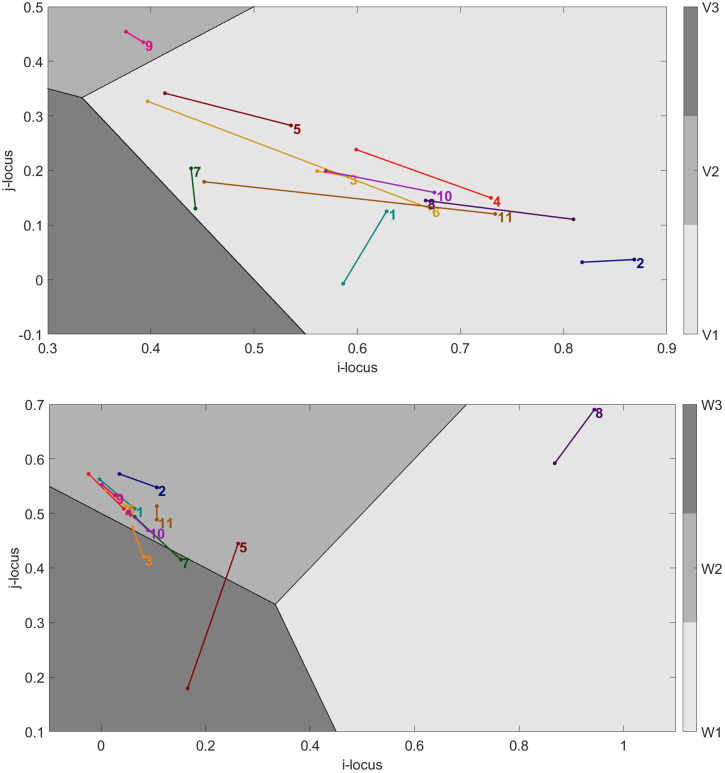
Dyads’ trajectories in the information spaces for the control of the centring torque τ_B_ (*Top* panel) and the forward torque τ_P_ (*Bottom* panel). In the information space related to **τ**_**B**_, the component variables are **V1**: ball’s outward acceleration, **V2**: negative of first derivative of angle between ball’s direction of motion and tangent to target midline, and **V3**: negative of board angle in the direction of the ball axis. In the information space related to **τ**_**P**_, the component variables are **W1**: ball’s outward velocity, **W2**: angular velocity of board in the direction of the ball axis, and **W3**: negative board angle in the direction of the ball axis. In the respective information spaces, the joint contribution of the three component variables is given by the compound informational variables ***V*** and ***W***, defined as: ***V*** = *i*(**V1**) + *j*(**V2**) + (1 – *i* – ***j*)**(**V3**) and ***W*** = *i*(**W1**) + *j*(**W2**) + (1 – *i* – ***j*)**(**W3**). The values of the weight coefficients *i* and *j* are used as coordinates (*i*, *j*) of the compound variable point in the two-dimensional information spaces. The coordinates of the compound variable are indicated in the spaces for Set 1 and Set 2 for each of the 11 coloured dyads, numbered at the Set 1 position. Finally, dominance regions are shaded in light, medium and dark grey to indicate where in the information space the contribution of the individual component variables to the information compound is most relevant.

The previous steps of our analyses allowed us to select three informational variables that were correlated with the torques applied around the ball axis and the perpendicular axis. The present subsection investigates how the use of these three informational variables evolved from the first set to the second set of the experiment, which is to say, how each of the 11 dyads changed in their use of the available information to learn how to successfully control the ball trajectory along the target. The learning behaviour of each dyad is represented by their respective trajectories in two information spaces, related to the torques **τ**_**B**_ and **τ**_**P**_. As described in more detail in the Materials and Methods section, such a path is obtained using two locations (i.e., one for Set 1 and one for Set 2) of a compound informational variable defined by a weighted linear combination of the three selected component variables. Fig 5 depicts the dyads’ trajectories in the information space related to torque **τ**_**B**_ (*Top* panel) and the information space related to torque **τ**_**P**_ (*Bottom* panel). In these information spaces, each of the three shaded regions (light, medium and dark grey) indicates the values of the weight coefficients *i* and *j* for which one of the component variables (**V1**, **V2** and **V3**; or **W1**, **W2** and **W3**, depending on the space considered) has a larger contribution to the informational compound (***V*** or ***W***) than the other component variables. Graphically, each point in a dyad’s trajectory corresponds to a compound variable with coordinates (*i*, *j*), positioned within a shaded dominance region that indicates which variable has the largest contribution to the compound.

In both spaces, dyads seemed to navigate in similar ways in terms of information usage. They tended to depart from the same region in Set 1 and to head in the same direction in Set 2. In other words, the information that was used and the process of changing in information usage from Set 1 to Set 2 were relatively homogeneous across dyads.

In the information space related to **τ**_**B**,_ the dominant component of the information compound was the ball’s outward acceleration. This was the case for all dyads, with the exception of the dyad with the best performance (i.e., Dyad 9; Appendix A). However, the locations in Set 2 revealed that the dyads tended to move in the direction of the other information regions. For further insights, correlations between the *i* and *j* coordinates (i.e., determining the use of information) and the number of crossings (i.e., determining performance) were computed for both sets, investigating whether the dyads’ locations in the information space were related to their levels of performance. Although this correlation between information locus and task performance was not significant for Set 1 (**p* *> .05), statistical significance was observed for Set 2 (**p* *< .05). More specifically, better performance in Set 2 was correlated with a lower *i* coefficient (**r* *= – 0.66) and a higher *j* coefficient (**r* *= 0.73). Given the equation ***V*** = *i*(**V1**) + *j*(**V2**) + (1 – *i* – *j*)(**V3**) that determines the relative contributions of the components, this indicates that an increase in performance was correlated with more reliance on the derivative of the angle between the ball direction and the tangent to the target path (**V2**) and less reliance on the ball’s outward acceleration (**V1**). As illustrated, the derivative of the angle between the ball direction and the tangent to the target path was most clearly used by the dyad with the best performance (i.e., Dyad 9; Appendix A) and got increasingly used by dyads with good performance in Set 2 (e.g., Dyads 3–6; Appendix A). In contrast, the dyads with the lowest performance (i.e., Dyads 2 and 8; Appendix A) exhibited trajectories that did not get close to the regions in the space with informational variables that were most useful for the control of the ball trajectory.

In the information space related to **τ**_**P**,_ the general tendency was to increasingly move in the direction of information related to the derivative of the board angle that lifts the ball axis. In other words, the rate of change of the board inclination that draws the ball towards the board centre was an increasingly important part of the information used to control the force applied to move the ball forwards. Additionally, Dyad 5 increasingly relied on the board angle that moves the ball forwards to regulate the torque responsible for steering the ball in that same direction. Even though no significant correlation between information usage and task performance was observed to further explain the control of torque **τ**_**P**_, Dyad 8 (i.e., the dyad with the lowest performance; Appendix A) was located in another region than the cluster that represented the overall location of the other dyads in the information space. Indeed, the torque **τ**_**P**_ exerted by this dyad was most closely related to pure ball kinematics (i.e., the outward velocity of the ball), combining this information to a lesser extend with the board kinematics than the other dyads.

## 4. Discussion

In the present paper, the effects of practice on the performers’ production of movements and use of information in a joint-action task were analysed. To do so, we used the concept of information space that was developed in the context of the direct learning theory [12]. More broadly, a major objective of this study was to discuss the extent to which the methodological concept of information space could have practical applications for learning assessment in clinical settings.

### 4.1. Practice effects: less task-irrelevant movements, more task-relevant information

As predicted by our first hypothesis, overall task performance increased with practice. However, not all participants demonstrated such improvement. At first glance, this lack of progress might suggest a ceiling effect associated with the development of expertise in the game. Yet, all participants were novices undertaking a novel and relatively complex multi-joint dyadic supra-postural task, demanding coordination, precision, and balance. Considering these factors, the 30-trial experiment may not have been sufficient to allow the attunement to functional perception-action couplings for those dyads whose performance did not improve – namely, Dyads 2 and 11. Additional research is necessary to inspect whether prolonged practice would enhance task performance for all dyads. Furthermore, while Dyads 2 and 11 also navigated in information spaces to detect more task-relevant information (i.e., *education of attention*), their performance might have been limited by a lack of coupling between information and action parameters (i.e., *calibration*) [12]. This highlights another limitation of our methodology: while we focused on the informational variables ***I*** that specify the task-relevant action parameters ***A*** (i.e., torques **τ**_**B**_ and **τ**_**P**_), individual differences and changes of the calibration function ***f*** that couples information and movement in the relation ***A*** = ***f*** (***I***) were not addressed. This currently missing aspect of motor learning – namely, how the exerted torques are related to the informational variables – could potentially be explored through calibration spaces [12,14]. At present, nevertheless, our information-space analysis revealed that information usage during practice – and overall performance improvement – was remarkably homogeneous across dyads, providing valuable insight into how information drives motor learning within an original joint-action task designed to resemble real-life dyadic interactions.

At the behavioural level, we expected that dyads would come to produce more task-relevant movements and less task-irrelevant movements after practice. Task-relevant movements can be defined as board movements that facilitate the control of the ball trajectory. Arguably, these are the rotations around the horizontal axes of rotation (i.e., the roll and pitch), because such rotations cause inclinations of the board that make the ball roll along the board. Other types of board movements are less directly related to the movement of the ball and are hence referred to as task-irrelevant movements. To test our hypotheses concerning task-relevant and task-irrelevant movements, we analysed the variability in translational and rotational movements of the board in all three dimensions, over all 30 trials. Our findings could not verify the hypothesis that predicted an increased use of task-relevant movements with practice: neither the roll nor the pitch variability was significantly affected by the amount of practice. In contrast, the variability in all other movement variables significantly decreased. To get more specific, less and less board translational movements were observed in all three dimensions, and the variability in the board rotations around the vertical axis (i.e., the yaw) was also reduced. We believe that the variability in these types of movement was reduced because these movements are less relevant for the to-be-performed task. Beyond being less relevant, they may also impede task performance by destabilizing the performers playing on an unstable surface, due to pushing and pulling forces transmitted through the hand-held board ring. In addition, producing translational movements along the vertical axis (i.e., up-down movements) is undesirable, because such movements can result in excessive board heights. This not only destabilizes players by affecting their centre of mass but also hinders the detection of information related to the ball and target locations. To sum up, dyads produced less pronounced task-irrelevant (or task-impeding) movements with practice, rather than more pronounced roll and pitch rotations responsible for guiding the ball towards the target.

Instead of improving by increasing the amount of roll and pitch rotations, it seems that participants were attuning to information that allowed them to better exploit the complex interplay among the actively controlled forces and the reactional and/or passive forces that are involved in the task, including the gravitational force. As a possible example of this, note that nearly all the trajectories in the information space related to **τ**_**P**_ (i.e., the torque around the ball axis that drives the ball forwards) demonstrate an increased use over practice of the angular velocity of the board in the direction of the ball axis (i.e., the rate of change of the board inclination that drives the ball towards the centre). The positive correlation that was observed between this centring angular velocity of the board and the exerted forward torque (**τ**_**P**_) can be explained by the importance of maintaining the ball’s momentum while steering it along the target. When the centring rotation is stronger (i.e., the angular board velocity is higher), a greater **τ**_**P**_ may be required to oppose a movement that spirals inwards, and hence to achieve a sustainable circular movement. Therefore, the angular velocity responsible for centring the ball may act at least partly as an informational variable, signalling how to increase **τ**_**P**_ to execute a more curved or less curved trajectory. In their ball-and-beam task – which can be viewed as a lower-dimensional version of our ball-and-board task – Hafkamp and colleagues [18] also demonstrated that dyads could learn how to exploit the gravitational forces in the system by producing larger beam inclination amplitudes.

The information specifying the roll-pitch relations, as discussed in the previous paragraph, seemed to be exploited by nearly all dyads for the control of **τ**_**P**_. The exception was the dyad with lowest performance, which was located in the region of the information space with a larger contribution of the ball’s outward velocity. This may suggest that this low-performance dyad would merely react to ball’s outward motion to control the torque that drives the ball forwards, rather than establishing and taking advantage of a more optimal roll-pitch coupling. To regulate **τ**_**B**_, dyads with lower performance also relied significantly more on a variable related to the outward movement of the ball (i.e., the second derivative of the outward movement) than dyads with higher performance. For both torques, therefore, the learning of the task implied a reduced reliance on the derivatives of the pure outward ball movement. For the control of **τ**_**B**_, dyads seemed to navigate in the direction of the board angle lifting the ball axis and the derivative of the angle between the ball’s direction of motion and the tangent of the target midline, in most cases without reaching the regions where these variables could be argued to be the dominant component variables.

In summary, even though our joint-action task does not seem to have been fully mastered at the end of the session, dyads minimized the production of less relevant and impeding movements with practice, and they increasingly relied on information that specified the board roll-pitch coupling and the ball trajectory in relation to the target. Although precise interpretations are complex, we believe that the observed movements in the information spaces indicate an attunement to variables that allows dyads to better exploit the multifaceted relations between the different forces relevant to the ball-and-board system. As an additional contribution, our results demonstrate that information-space analyses can be applied to a task that is performed by a pair of individuals (i.e., by a dyad), implying the control of many degrees of freedom. In previous studies, this type of analysis was only applied to apparently simpler tasks that were performed by individuals [14].

### 4.2. Information spaces to assess perception-action: clinical implications

In the present paper, information spaces illustrated how dyads exploited the information available in ambient energy arrays to control the movements required to successfully perform the task. To put it differently, this methodological tool was particularly useful to depict how perception-action couplings evolve with practice. Because it may be a challenge for therapists to assess and strengthen altered perception-action couplings in patients with motor disorders, we would like to discuss how the methodology employed in the present study might have practical implications in rehabilitation.

Pioneering attempts to apply the ecological dynamics approach of motor learning to motor disorders originated from the 1980-90s [1,19–21]. Several tools and practical guidelines were accordingly provided to bridge the translation gap between research and clinical practice. Among them, action-scaled measures have been suggested to inspect mismatches between the actor and environment, enabling the design of interventions based on actor-environment units, rather than on external biological standards [22]. Such mismatches can thereby be studied using the Gibsonian concept of affordances, as well as through biomechanical models that consider both the effectiveness and energy cost of the movement solutions exhibited by the patient [23]. Both assessment methods arise from the assumption that non-standard movement patterns are not necessarily pathological, and that impairments are just one type of constraint added to the self-organized actor-environment system, which seeks an optimal solution to the motor problem it faces. It is then the therapist’s mission to estimate whether these patterns may lead to undesirable long-term consequences. If they do, the therapist can intervene to guide the system towards more adaptative movement solutions, using techniques that assess coordination dynamics. In this context, re-shaping performer, environmental or task constraints is necessary to destabilize the current dysfunctional solution and help the discovery of a healthier one [1,2,20–26]. Methodological recommendations have been proposed to map and act on the patient’s dynamical landscape – a topology of the behavioural repertoire – towards more or less stable solutions [1,20,21,27]. Tools derived from a dynamical view of motor learning thus allow tracking behavioural changes, such as the emergence of new coordination patterns or a greater ability to switch between patterns, taking advantage of the body system redundancy in a given context of constraints and possibilities.

In complement, “coordination profiling” with clusters of performance has been suggested as a qualitative technique to assess the variability of movement patterns in rehabilitation motor tasks [28]. In the same vein, we propose in the present study how such clusters of performance could be represented in information spaces, to assess how patients exploit the available information during the re-learning of a motor task. Theoretically, the notion of information space could in some sense be seen as complementary to dynamical landscapes, because dynamical landscapes traditionally place much emphasis on the action side of perception-action couplings while information spaces emphasize the perceptual side. Practically, assessing performance with this direct learning tool could provide directions for the therapist to adequately manipulate informational constraints to foster the emergence of adaptative movement solutions. In addition to the assessment of performance with information spaces, a for-the-moment more speculative way to actually achieve quicker movements through such spaces could consist in enhancing the information that guides the learning itself with purposefully chosen variability in the practice conditions [29].

To better support our main point, we used our joint-action task – where players jointly engage in a ball-and-board game while standing on an unstable surface – as an example of how re-learning perception-action can be assessed in patients with lower limb injuries. To reduce the risk of ACL (re)injury, it was recommended to maximize movement exploration through constraints manipulation, rather than prescribe specific movement patterns [30,31]. Because ACL-deficient patients predominantly rely on visual information to control upright posture on an unstable surface [32], it would be interesting to observe in information spaces how they adapt the information-movement couplings to successfully perform our joint-action task. Task constraints could then be adjusted accordingly to guide them in the re-learning of perception-action. We encourage future research to investigate the potential benefits of using information spaces with pathological populations in rehabilitation settings.

In conclusion, the present study provides evidence that the practice of a novel joint-action task can be described and assessed as a process that implies changes in movement production and information usage. Although no increase in task-relevant movements was observed with practice, dyads demonstrated less pronounced movements that might impede task performance, while increasingly relying on informational variables that allow them to take advantage of the complex interplay of the different types of forces that are relevant in the ball-and-board system. Importantly, we demonstrated how information spaces could be used to address questions related to motor learning in novice players, who need to learn the perception-action couplings that are required for a new joint-action task. Finally, we discussed how the present methodology may be useful in clinical settings, where patients with motor disorders need to re-learn perception-action couplings, which should then be assessed by therapists to better shape their interventions.

## Supporting information

S1 Data Appendix A. (DOCX)

S2 Data Raw Data. (ZIP)
