# Supplementary figures and images for: How to jointly control a ball trajectory on a moving board: Methodological insights into Motor Learning and Rehabilitation
Source: PLoS One. 2025 Oct 15;20(10):e0334588. doi: 10.1371/journal.pone.0334588 (PMC12527165; doi:10.1371/journal.pone.0334588)

**Appendix A**

**Evolution of the performance scores from Set 1 to Set 2 in all dyads.**


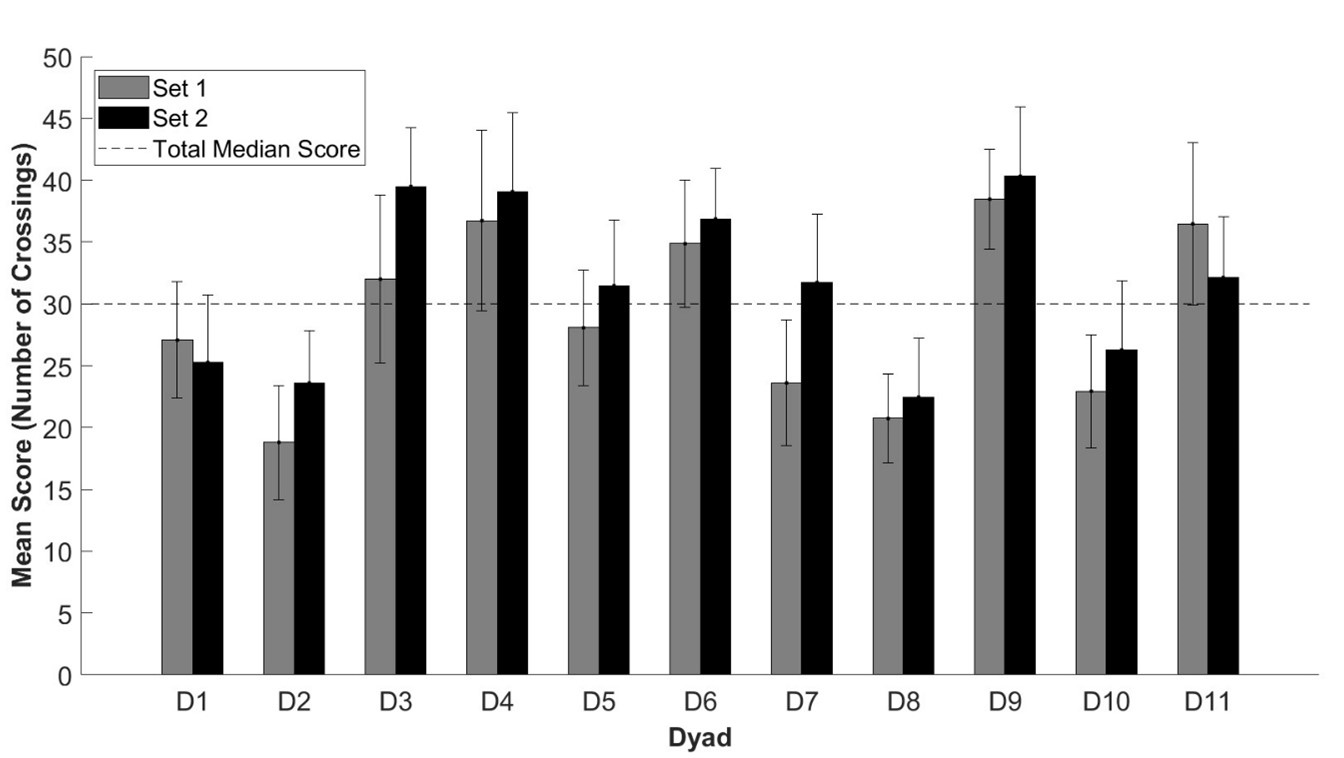

Supplement: S1 Data — (DOCX) [file pone.0334588.s001.docx]
